# Application of Dual Mask for Postoperative Respiratory Support in Obstructive Sleep Apnea Patient

**DOI:** 10.1155/2013/321054

**Published:** 2013-04-10

**Authors:** Jahan Porhomayon, Gino Zadeii, Nader D. Nader, George R. Bancroft, Alireza Yarahamadi

**Affiliations:** ^1^VA Western New York Healthcare System, Division of Critical Care and Pain Medicine, Department of Anesthesiology, State University of New York at Buffalo, School of Medicine and Biomedical Sciences, VA Medical Center, Room 203C, 3495 Bailey Avenue, Buffalo, NY 14215, USA; ^2^University of Iowa, Mason City Cardiology, Mason City, IA 50401, USA; ^3^VA Western New York Healthcare System, Division of Cardiothoracic Anesthesia and Pain Medicine, Department of Anesthesiology, State University of New York at Buffalo School of Medicine and Biomedical Sciences, Buffalo, NY 14215, USA; ^4^Department of Anesthesiology, State University of New York at Buffalo, School of Medicine and Biomedical Sciences, Buffalo, NY 14215, USA; ^5^Director of Mercy North-Iowa Neurology and Sleep Laboratory, University of Iowa, Mason City Neurology, Mason City, IA 50401, USA

## Abstract

In some conditions continuous positive airway pressure (CPAP) or bilevel positive airway pressure (BIPAP) therapy alone fails to provide satisfactory oxygenation. In these situations oxygen (O_2_) is often being added to CPAP/BIPAP mask or hose. Central sleep apnea and obstructive sleep apnea (OSA) are often present along with other chronic conditions, such as chronic obstructive pulmonary disease (COPD), congestive heart failure, pulmonary fibrosis, neuromuscular disorders, chronic narcotic use, or central hypoventilation syndrome. Any of these conditions may lead to the need for supplemental O_2_ administration during the titration process. Maximization of comfort, by delivering O_2_ directly via a nasal cannula through the mask, will provide better oxygenation and ultimately treat the patient with lower CPAP/BIPAP pressure.

## 1. Introduction

Obstructive sleep apnea (OSA) is a complex medical disorder, characterized by repetitive upper airway collapse during sleep. The disease affects individuals of all ages and predisposes to multiple comorbidities, including increased risk of cardiovascular disease [1].

Perioperative apneas appear to be multifactorial in nature. Sedatives and anesthetics have been shown to decrease pharyngeal muscle tone and therefore predispose to apnea [2]. Meanwhile, the patient's normal arousal responses and reflexes are also compromised by anesthetics [3]. This predisposes to apneic episodes which can be more severe than those associated with natural sleep.

While many patients present for surgery with undiagnosed OSA, it is currently recommended that patients who receive ambulatory CPAP preoperatively should continue to have CPAP administered in the perioperative period. Otherwise, the optimal management of OSA in the perioperative period has yet to be elucidated [4].

## 2. Case Report

A 51-year-old obese male, with a history of daytime fatigue, presented to the anesthesia holding area for urgent appendectomy. He had previously undergone a sleep study several months before with apnea/hypopnea index (AHI) of 35 and a maximum desaturation to the low 60's. Patient vital signs included a blood pressure of 140/85 mm/Hg, heart rate of 95 beats per minute and respiratory rate of 16 per minute with a temperature of 38 centigrade. His pulse oximetry (SaO_2_) reading was 91% with 2 liters/minute of nasal oxygen flow. Chest radiography did not show any pathology.

**Figure 1:**
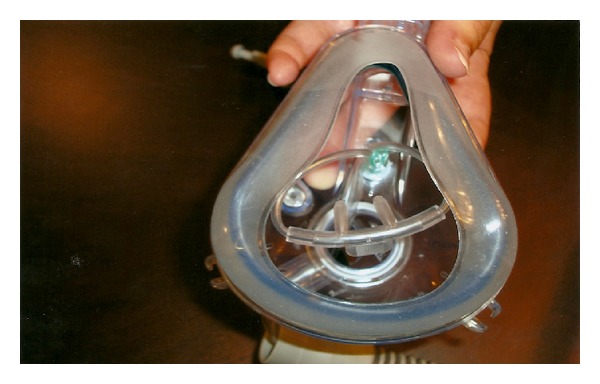
Dual mask is easy to apply with inner nasal cannula.

**Figure 2:**
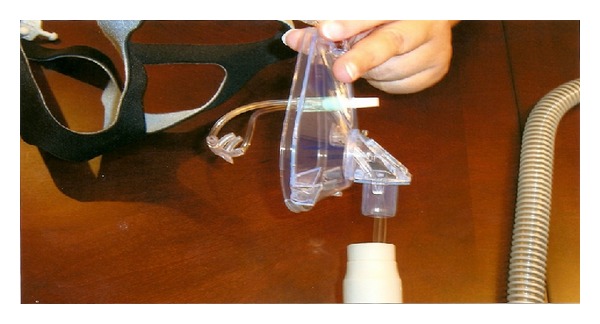
Lateral view of dual mask, nasal cannula can be directly applied to the patient nostril and eliminate dilution effect secondary to leak.

He was brought to operating room, and anesthesia was induced with propofol and succinylcholine in a rapid sequence technique. The trachea was intubated with the aid of a GlideScope. Anesthesia was maintained with mixtures of oxygen/air/desflurane/fentanyl. The patient was extubated without difficulty and was transported to the PACU on supplemental O_2_ via mask with SaO_2_ of 90%. On arrival to PACU and after the first set of vital signs, his SaO_2_ dropped to 88%. As CPAP was applied, he immediately started to have central apneas. Therefore, we started BIPAP therapy with O_2_ concentration of 50%. Because the patient was a major mouth breather with full beard, we continued to have difficulty achieving appropriate tidal volumes. The PACU nurses tried multiple full-face masks with or without a chin strap, but low tidal volume alarms continued. At BIPAP pressures of 15/11 cm H_2_O and O_2_ flow of 4 liters/minutes, we could not improve the saturation. The nurses were unable to stabilize saturations due to numerous central apneas. We then decided to use the dual mask (Figures 1 and 2) with 2 liters/min of O_2_ via nasal cannula. We set the BIPAP machine to pressure of 8/4 cm H_2_O and were finally able to adjust the pressure to 12/6 cm H_2_O with SaO_2_ of 92% and adequate tidal volume.

## 3. Discussion

The potential for upper airway obstruction remains high postoperatively because of the effects of residual anesthetics, sedatives, and opioids. During the postoperative period, patients with severe obstructive sleep apnea are at increased risk for respiratory and cardiopulmonary complications [5]. If a patient has his or her own CPAP device, it should be available in the recovery room for use immediately upon emergence from anesthesia. Under no circumstances should the patient be left unattended [3, 6]. Close observation is required until stability is established.

The use of higher CPAP/BIPAP pressure can lead to higher leakage, increased nasal drying or congestion, pressure sores on the bridge of the nose, difficulty exhaling, and higher levels of machine noise [7, 8]. Clearly, these are disadvantageous, and lower pressures are preferred.

In current clinical practice O_2_ is added to CPAP/BIPAP mask usually from a small hole into the mask or to CPAP/BIPAP hose. Hence, the added O_2_ becomes diluted by CPAP/BIPAP flow and is also subject to leakage [7]. Patients rarely get the full benefit of supplemental O_2_.

Adding O_2_ directly to the patient's mask is not practical because it can easily come disconnected when the patient changes position. This is a common source of patient complaints. The pressure can also affect the triggering function of the ventilator [9]. Another disadvantage is having two separate tubes on the bed and two separate machines at the bedside.

Combining the O_2_ compressor and CPAP/BIPAP machine as well as combining the CPAP/BIPAP hose and the O_2_ hose (dual tube) to occupy less space can provide better convenience. Connecting an adjustable cannula to a full-face or nasal mask and delivering supplemental O_2_ directly to the nostrils will provide higher O_2_ saturation during inhalation with the help of CPAP/BIPAP pressure [7].
